# The Feasibility of Implementing Interprofessional Collaboration–Based Telecare Services for Patients With Tuberculosis: A Mixed Methods Study From Hospital Insight

**DOI:** 10.1155/ipid/2070413

**Published:** 2026-04-16

**Authors:** Devi Ristian Octavia, Andi Hermansyah, Yunita Nita, Fadli Asmani, Ibrahim Abdullah

**Affiliations:** ^1^ Doctoral Program Pharmaceutical Science, Faculty of Pharmacy, Universitas Airlangga, Surabaya, East Java, Indonesia, unair.ac.id; ^2^ Faculty of Health Sciences, Universitas Muhammadiyah Lamongan, Lamongan, East Java, Indonesia; ^3^ Department of Pharmacy Practice, Faculty of Pharmacy, Universitas Airlangga, Surabaya, East Java, Indonesia, unair.ac.id; ^4^ Faculty of Pharmacy, Innovative Pharmacy Practice and Integrated Outcomes Research (INACORE), Universitas Airlangga, Surabaya, East Java, Indonesia, unair.ac.id; ^5^ Research Center in Advancing Community Healthcare (REACH), Universitas Airlangga, Surabaya, East Java, Indonesia, unair.ac.id; ^6^ School of Pharmacy, Management and Science University, Shah Alam, Selangor, Malaysia, msu.edu.my

**Keywords:** digital health, health care, interprofessional collaboration, telemedicine, tuberculosis

## Abstract

**Background:**

Tuberculosis (TB) remains a major global health challenge and is a leading cause of mortality in low‐ and middle‐income countries. Indonesia continues to record one of the world’s highest TB burdens. Persistent gaps in treatment adherence and continuity of care remain, despite various national initiatives. Achieving the national TB elimination target requires innovative, patient‐centered approaches. These must be supported by interprofessional collaboration (IPC) and digital health interventions. This study aimed to assess the feasibility of implementing telecare‐based IPC services to optimize TB management in hospital settings.

**Methods:**

A mixed‐methods exploratory sequential design was employed, comprising a quantitative cross‐sectional survey followed by qualitative focus group discussions (FGDs). The study was conducted at Husada Prima Hospital, Surabaya, a regional TB referral center in Indonesia. In the quantitative phase, 77 healthcare professionals, including doctors, pharmacists, and nurses, completed the validated Indonesian version of the collaborative practice assessment tool (CPAT). Data were analyzed using the Kruskal–Wallis and Mann–Whitney *U* tests to examine differences in IPC perceptions across professional groups. The qualitative phase involved FGDs with 12 participants representing doctors, nurses, pharmacists, and hospital management. Discussions were transcribed verbatim and analyzed thematically to identify opportunities, barriers, and implementation strategies for telecare‐based IPC. Rigor was maintained through data triangulation, participant validation, and information saturation.

**Result:**

Quantitative analysis identified significant interprofessional differences in perceptions of IPC, particularly regarding team barriers (*p* = 0.003), coordination and division of roles (*p* = 0.008), decision‐making and conflict management (*p* = 0.025), and mission, goals, and objectives (*p* = 0.034). Qualitative analysis produced four major themes and 13 subthemes, encompassing opportunities, barriers, implementation strategies, and organizational support for telecare‐based IPC. While participants expressed optimism about the model’s potential, they also highlighted challenges, including unclear task delineation, patient skepticism toward digital communication, and the absence of standard operating procedures. Integration of quantitative and qualitative findings indicated that clear leadership structures, sufficient digital infrastructure, and robust professional collaboration are essential for successful implementation.

**Conclusion:**

Telecare‐based interprofessional collaboration is feasible and has the potential to enhance TB care in hospital settings. However, successful adoption depends on adequate infrastructure, well‐prepared human resources, and supportive policies. The model is adaptable to diverse healthcare contexts but should be tailored to local capacities and patient characteristics to achieve optimal outcomes.

## 1. Introduction

Tuberculosis (TB) remains a major global health concern and is a leading cause of death from infectious diseases. Despite progress in prevention and treatment, TB continues to cause high rates of illness and death, with 10.6 million new cases and 1.3 million deaths reported in 2023 [1]. The ongoing prevalence of TB results from complex biological, social, and health system barriers that impede case detection, treatment adherence, and continuity of care, especially in low‐ and middle‐income countries (LMICs) [2]. Meeting the World Health Organization (WHO) End TB Strategy targets of a 90% reduction in incidence and a 95% reduction in mortality by 2035 requires both biomedical innovation and coordinated multisectoral, interprofessional strategies that address the full spectrum of patient needs [3].

Interprofessional practice is fundamental to patient‐centered TB care, delivering comprehensive treatment through coordinated medical management, psychological support, and social empowerment for patients and their family care partners. Collaboration among physicians, nurses, pharmacists, and community health workers enables integrated interventions throughout the TB care continuum [4, 5]. This approach improves treatment adherence, reduces loss to follow‐up, and enhances quality of life [6–8]. Such collaboration is essential in managing chronic infectious diseases such as TB, where extended treatment regimens and social stigma may negatively affect outcomes if care is fragmented [9–11].

To address the need for innovation, the WHO launched the End TB Strategy and the Global Task Force on Digital Health for TB. It later updated in 2022 to encourage the adoption of digital tools in TB care and prevention [12, 13]. This strategy promotes the integration of validated digital health tools, such as telecare, mobile applications, and electronic monitoring, into routine TB services and public health surveillance. Telecare in particular can expand TB care by enabling remote consultations, video‐observed therapy (VDOT), and ongoing patient engagement [14–16]. Advances in healthcare technology now make it possible to provide telecare services that can improve treatment support for TB patients. Optimism and innovation play key roles in shaping patients’ willingness to adopt telecare solutions. Previous research has shown that both patients and healthcare providers find telecare services acceptable. Moreover, telehealth offers time efficiency and cost‐effectiveness [17, 18].

Although telecare has improved patient outcomes, service efficiency, access to specialized care, and expert guidance in various settings, its implementation in LMICs faces significant barriers. These include unreliable internet connectivity, limited digital literacy, insufficient healthcare worker training, inadequate funding, and a lack of supportive policy frameworks [19]. Addressing these structural and systemic challenges is essential for achieving equitable access and sustainable integration of digital health innovations in TB management.

Indonesia ranks second globally in TB disease burden, with over one million cases and approximately 134,000 deaths annually [1]. Despite national programs for case detection and treatment, significant gaps remain in treatment adherence and continuity of care. While telecare may address these challenges, the feasibility of telecare‐based interprofessional collaboration (IPC) for TB management in Indonesia has not been comprehensively studied. Existing evidence does not adequately demonstrate how integrating digital health tools with team‐based care can overcome these barriers or improve patient engagement, adherence, and outcomes, indicating a critical research gap [2].

This study evaluates the feasibility of telecare services based on IPC for TB patients. Assessing the acceptability, practicality, and potential effectiveness of this approach will generate insights to improve TB services in resource‐limited settings and advance progress toward End TB targets.

## 2. Materials and Methods

### 2.1. Study Design

This study employed a mixed‐methods exploratory sequential design, integrating quantitative and qualitative methods to thoroughly assess the feasibility of implementing a telecare‐based IPC service for TB management. The design featured two sequential phases: First, a quantitative cross‐sectional survey to assess healthcare professionals’ perceptions of IPC, followed by a qualitative phase using focus group discussions (FGDs) to identify contextual barriers, facilitators, and implementation strategies.

### 2.2. Context and Setting

The study was conducted at Husada Prima Hospital in Surabaya, Indonesia. Previously a specialized lung hospital, it currently serves as a regional referral center for TB in East Java, treating 300 to 400 TB patients monthly in 2024. A multidisciplinary team, including physicians, nurses, and pharmacists, manages TB care. However, operational challenges persist, such as overlapping roles, inadequate communication, and unclear task delineation.

### 2.3. Quantitative Phase

#### 2.3.1. Study Design and Population

The quantitative phase utilized a cross‐sectional design involving healthcare workers directly engaged in TB patient care at Husada Prima Hospital. The total population consisted of 105 health workers, including physicians, nurses, and pharmacists.

Sample size was determined using the proportion formula:

(1) n=z2 p 1−pd2 N−1+Z2 p 1−p.N,

where *Z* represents the standard normal deviation (1.96 for 95% confidence), *p* is the estimated proportion (0.5), *d* is the desired precision (0.05), and *N* is the total population size 105.

Stratified random sampling based on professional categories was employed, resulting in the inclusion of 77 respondents: (1) doctors: 13 respondents, (2) nurses: 60 respondents, and (3) pharmacists: 4 respondents.

#### 2.3.2. Inclusion and Exclusion Criteria

Inclusion criteria included (a) registered healthcare professionals (physicians, nurses, and pharmacists) directly involved in TB care, (b) minimum of 1 year of professional experience, and (c) willingness to participate in the study and provide informed consent. Exclusion criteria comprised participants who withdrew during the study or submitted incomplete responses.

### 2.4. Data Collection Instrument

Data were collected using the Indonesian version of the collaborative practice assessment tool (CPAT), a questionnaire designed to assess teamwork among healthcare providers and previously validated in a study with 63 respondents (Pearson correlation 0.5–0.9; Cronbach’s alpha = 0.970) [20]. The CPAT consists of eight domains comprising 53 items: (1) relationships among team members (9 items); (2) team barriers to collaboration (5 items); (3) team relationships with the community (4 items); (4) coordination and role division (14 items); (5) decision‐making and conflict management (2 items); (6) leadership (5 items); (7) mission, goals, and objectives (9 items); and (8) patient involvement (5 items).

Responses were rated on a five‐point Likert scale ranging from “*strongly disagree*” to “*strongly agree*.” Data collection was conducted through a self‐administered online survey distributed via institutional email and messaging applications. Completion of the questionnaire was considered to constitute informed consent. The survey was available for two weeks and took approximately 10–15 min to complete.

### 2.5. Data Analysis

Quantitative data analysis was performed using IBM SPSS Version 25.0 (IBM Corp., Armonk, NY, USA). Descriptive statistics summarized demographic characteristics and domain‐level responses. The Kruskal–Wallis test was applied to examine differences in perceptions of IPC across professional groups, with post hoc analyses using the Mann–Whitney *U* test to identify pairwise differences. Statistical significance was defined as *p* < 0.05.

### 2.6. Qualitative Phase

#### 2.6.1. Study Participants and Sampling

The qualitative phase collected in‐depth perspectives from healthcare professionals and hospital management regarding the feasibility of telecare‐based IPC. Purposive sampling was used to ensure diversity in profession, age, gender, education, and TB care experience. Twelve informants participated, comprising physicians, nurses, pharmacists, and hospital administrators.

Participants were recruited through the hospital’s Research and Development Division. Study information and informed consent documents were distributed at least 1 week prior to participation. No participants had a pre‐existing relationship with the researchers.

#### 2.6.2. Data Collection Procedure

Data were collected through FGDs conducted in Indonesian, each moderated by a trained facilitator. The research team consisted of doctoral students in Pharmaceutical Sciences under the supervision of two academic experts in pharmacy practice. The FGDs aimed to elicit diverse perspectives on IPC and telecare implementation.

Discussion topics included (a) current practices of IPC in TB management, (b) perceived feasibility and benefits of telecare for improving medication adherence, (c) barriers and enablers to implementing telecare‐based IPC, and (d) strategies to operationalize IPC‐driven telecare within hospital workflows.

Each FGD lasted approximately 90–120 min and was audio‐ and video‐recorded with participant consent. An assistant researcher documented key discussion points and nonverbal cues. Participant identities were anonymized, and all data were stored securely.

#### 2.6.3. Data Trustworthiness

Trustworthiness was ensured through multiple strategies, as follows:

• Credibility is achieved through member checking, where participants reviewed and verified the accuracy of transcriptions and key findings.
• Dependability was maintained through a detailed audit trail documenting methodological decisions, coding processes, and analytical interpretations.
• Confirmability was ensured through team discussions and consensus‐building to minimize researcher bias.
• Transferability was enhanced by providing a comprehensive description of the study setting, participants, and FGD procedures.

### 2.7. Data Analysis

Verbatim transcriptions were analyzed thematically using four main stages:

1. Familiarization—reading and rereading transcripts to understand data context;
2. Developing a thematic framework—identifying meaningful units related to research objectives;
3. Coding and categorization—assigning labels and organizing codes into themes and subthemes;
4. Interpretation—synthesizing relationships among themes to generate insights.

Field notes were integrated during coding to enhance contextual understanding. Emerging themes were translated into English and reviewed by all researchers. Discrepancies were resolved through team discussions until consensus was achieved.

Quantitative and qualitative findings were integrated during the interpretation phase to provide a comprehensive understanding of IPC readiness, contextual barriers, and opportunities for telecare implementation. Integration followed a convergent interpretation approach, with qualitative insights that explained and expanded upon quantitative findings. These insights informed recommendations for a telecare‐based IPC model for TB care.

### 2.8. Ethical Considerations

All participants provided informed consent prior to participation. Confidentiality and anonymity were strictly maintained throughout the study. Data were securely stored and accessible only to the research team. The study received approval from the Health Research Ethics Committee of Husada Prima Hospital Surabaya (Document No. 045/008.06/EC/KEPK/2024).

## 3. Result

The research results indicate that more than half of the professionals in the fields of medicine, pharmacy, and nursing were female. Most doctors were in the older age group (46–55 years), pharmacists were in the middle‐age group (36–45 years), and nurses had a more even age distribution. Most respondents were in the 1–5 years of practice experience category. No significant differences were found in the distribution of gender, age, or length across the three professions (*p* values > 0.05), as shown in Table 1.

**TABLE 1:** Characteristics of healthcare worker respondents involved in TB patient care (*n* = 77).

		***n* (%)**				
**Characteristics**	**Category**	**Total (*n* = 77)**	**Doctor (*n* = 13)**	**Pharmacist (*n* = 4)**	**Nurse (*n* = 60)**	**p** **value**
Gender	Male	29 (37.66)	4 (30.77)	1 (25.00)	24 (40.00)	0.133^a^
	Female	48 (62.33)	9 (69.23)	3 (75.00)	36 (60.00)	

Age	17–25	3 (3.9)	1 (7.69)		2 (3.33)	0.063^a^
	26–35	28 (36.36)	3 (23.08)	1 (25.00)	24 (40.00)	
	36–45	30 (38.96)	2 (15.38)	3 (75.00)	25 (41.67)	
	46–55	14 (18.18)	6 (46.15)		8 (13.33)	
	> 55	2 (2.6)	1 (7.69)		1 (1.67)	

Length of practice	1–5	26 (33.76)	5 (38.46)	1 (25.00)	20 (33.33)	0.097^a^
	6–10	19 (24.67)	4 (30.77)	2 (50.00)	13 (21.67)	
	11–15	11 (14.28)	2 (15.38)		9 (15.00)	
	16–20	10 (12.98)	1 (7.69)	1 (25.00)	8 (13.33)	
	> 20	11 (14.28)	1 (7.69)		10 (16.67)	

^a^Kruskal–Wallis’s test.

There was a significant difference (*p* < 0.05) in the perception of health workers regarding the practice of IPC, especially in the domain of (a) team barriers in collaboration where nurses faced more obstacles than doctors and pharmacists; (b) coordination and division of roles of which doctors showed more positive perceptions regarding coordination, while nurses showed the lowest perception; (c) decision‐making and conflict management where pharmacists showed better perception in conflict management than doctors; lastly, (d) mission, goals, and objectives in which doctors had higher perceptions than nurses regarding the clarity of these elements (Table 2).

**TABLE 2:** Test results of differences in perceptions of collaborative practice by profession.

Domain	Mean rank			*p* value^a^
	Doctor	Pharmacist	Nurse	
Relations between members	31.46	23.63	41.66	0.109
Team barriers in collaboration	20.81	31.25	43.46	0.003^∗^
Team relationship with community	50.54	36.63	36.66	0.097
Coordination and division of roles	55.46	46.13	34.96	0.008^∗^
Decision‐making and conflict management	27.96	50.50	40.63	0.025^∗^
Leadership	44.69	48.75	37.12	0.328
Mission, goals, and objectives	52.65	44.88	35.65	0.034^∗^
Patient involvement	45.54	55.25	36.50	0.126

^a^Wilcoxon test.

^∗^Significant difference.

The results in Table 3 indicate significant differences in views between doctors and nurses, particularly regarding collaboration and role division, which can affect teamwork effectiveness.

**TABLE 3:** Analysis of differences in healthcare workers’ perceptions across professions.

Domain	*p* value^a^		
	Doctor–nurse	Doctor–pharmacist	Nurse–pharmacist
Relations between members	0.130	0.602	0.104
Team barriers in collaboration	0.001^∗^	0.185	0.233
Team relationship with community	0.031^∗^	0.269	1.00
Coordination and division of roles	0.02^∗^	0.490	0.328
Decision‐making and conflict management	0.16	0.065	0.246
Leadership	0.223	0.445	0.338
Mission, goals, and objectives	0.12	0.327	0.361
Patient involvement	0.162	0.248	0.117

^a^Wilcoxon test.

^∗^Significant difference.

The FGD was conducted for 12 participants who were invited based on their expertise and experience in caring for TB patients in hospitals. The participants included specialist doctors, pharmacists, TB nurses, and pharmaceutical vocational staff. More than half of the participants (75%) were female, and most were of productive age, 35–55. The participants’ characteristics are presented in Table 4.

**TABLE 4:** Participant characteristics.

Characteristics	*f* (%)
*Background of the participants*	
Pulmonologist	3 (25%)
TB nurse	2 (16.67%)
Pharmacist	2 (16.67%)
Hospital management	4 (33.33%)
Pharmacy vocational staff	1 (8.33%)

*Gender*	
Male	3 (25%)
Female	9 (75%)

*Age (years old)*	
26–35	
35–45	5 (41.6%)
45–55	5 (41.6%)
> 55	2 (16.67%)

*Education*	
Diploma	2 (16.67%)
Bachelor	1 (8.33%)
Profession	2 (16.67%)
Master’s degree	7 (58.33%)

*Duration*	
Duration of the interview, in minute	100

We identified units of meaning related to the aims of this research. This means that units were coded inductively and classified into four main themes: opportunities, barriers, implementation, and support for telecare‐based IPC services (Table 5).

**TABLE 5:** Thematic analysis results of focus group discussion on the feasibility of interprofessional collaboration–based telecare for TB.

Theme	Subtheme	Quotations
Opportunities	Positive past experience	*“…I think this service is good because we (doctors) offer something more than the previous service. We have counseling at the hospital, then resume with the nurse, and finally at the pharmacy. When we take the medicine, we will also explain again how to take the medicine…” (PD, F, 05*) *“…do not let there be any overlap, because it is true, as stated when the patient arrives and is diagnosed, they are given education at the same time, etc., then when they reach the nurse too, then when they arrive at the pharmacy, when they receive the medicine, we also convey it…” (PA, M, 10)*
	Positive perception	*“…The thing is, we need to provide counseling actively, so the doctor gives the counseling himself, then the nurse at the front also usually does the resume” (PD, F, 05)* *“…education from the nurses, before going to the doctors, here you could say everyone is fussy about TB patients.” (PM, F, 03)*
	Infrastructure available	*“We have made several efforts, including here we have WhatsApp blasts, WhatsApp blast reminders for TB patients for control, and so on” (PM, F, 03)*
	Commitment to provide better services	*“If you look at this telecare service, I think in principle we could do it here” (PD, F, 05)*

Barriers	Task avoidance	*“…that is our challenge, and I think with this service it can be done, but that is just who is responsible for it and who it is, it has to be clear” (PD, F, 05)* *“…So, who do you think the responsible person is? Who is responsible? There is a third person between you, sir, or what…?” (PD, F, 06)* *“Who is in charge? Who is in contact? For example, if you apologize in the end, if only one person contacts you, what is this burden…” (PM, F, 02)*
	Patient skepticism	*“If, for example, the patient refuses, it is possible that on WA, he does not know who is calling, like when we get a call and do not know the name, we are too lazy to answer…” (PM, F, 02)*

Effective strategies	Collaboration between parties	*“We have a collaborative system because the government program is not just the responsibility of the hospital; we have collaboration with first-level health facilities, collaboration with community health centers, and if there are cadres, if they do not come, the cadres will visit them” (PM, F, 03)* *“The explanation must be correct…” (PM, F, 01)*
	Lack of protocol	*“…there are standards, general ones that are used to tell them they will understand that, we give limits…” (PD, F, 05)* *“…as long as we have someone in charge, the Standard Operating Procedures (SOP) are clear…” (PD, F, 05)* *“We ask for three cell phone numbers, at least three active ones, so that they can ring in front of your eyes, yes, at least three numbers that Like can contact at the bank, sis, and then addresses, I have to ask for ID card address and domicile address” (PP, F, 08)* *“As long as the officers have appropriate SOPs, the SOP is necessary” (PD, F, 05)*
	Career support	*“Support, the first companion is very important in this case. The patient’s family who accompanies, supports, most of them are left with their families” (PP, F, 08)*
	Priority for service recipients	*“The demographics of patients here are middle to lower class. “If it is lower middle class, there must be a lot of it. Why doesn’t he take medication regularly?” (PM, F, 03)* *“…but in reality, in the field, cellphone numbers do not necessarily mean that they are cellphones that can make WhatsApp” (PA, M, 10)*

**FIGURE 1:**
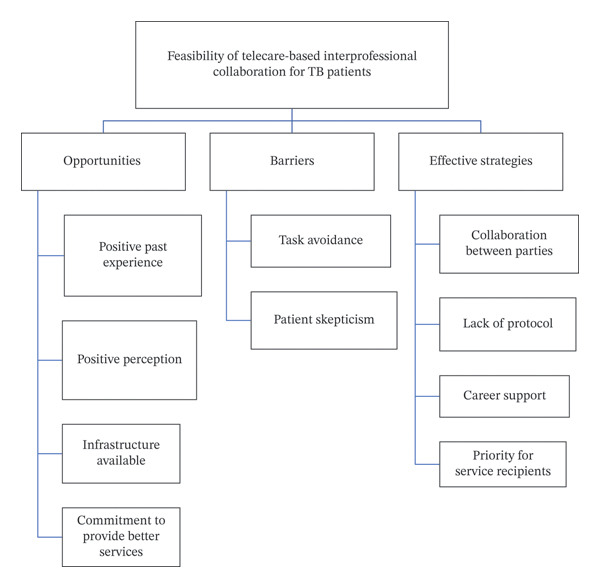
Themes and subthemes of the feasibility of interprofessional collaboration–based telecare in hospitals.

Opportunities consisted of two subthemes: the experience of health workers in TB elimination and the similarities in perceptions of TB education. Barriers consisted of three subthemes: organizational leadership, patient skepticism, and patient loss to follow‐up. Implementation consists of six subthemes: collaboration between parties, the need for guidance, patient characteristics, completeness of patient data, support systems, and the priority of service recipients. Meanwhile, the supporting theme has two subthemes: available infrastructure and commitment to implementing services. Figure 1 depicts the themes and subthemes.

### 3.1. Theme 1: Opportunities

Participants expressed optimism about integrating telecare with IPC. Previous collaborative experiences in TB counseling involving doctors, nurses, and pharmacists were identified as providing a foundation for adopting telecare‐supported services. Interprofessional communication was generally described as positive, and continuity of care was recognized as a key benefit.

*“I think this service is good because we [doctors] offer something more than before. We have counseling at the hospital, then continue with the nurse, and finally at the pharmacy.”* (PD, F, 05)

Participants also noted the presence of existing technological infrastructure, such as WhatsApp reminder systems, which could be utilized to support telecare interventions.

“*We already have WhatsApp blasts for TB control reminders. That can support the service.*” (PM, F, 03)

### 3.2. Theme 2: Barriers

Despite positive attitudes, participants identified several barriers. Role ambiguity and task avoidance were reported as ongoing challenges to effective interprofessional coordination. The need to clarify responsibilities within the telecare model was emphasized as a priority.

“*That is our challenge… who will be responsible for the service must be clear*.” (PD, F, 05)

Participants also noted that patient skepticism about remote communication could threaten engagement:

“*If the patient doesn’t know who is calling on WhatsApp, they may not answer. They feel uncertain.*” (PM, F, 02)

### 3.3. Theme 3: Implementation Strategies

Eager to overcome barriers, participants shared actionable strategies to propel telecare‐based IPC forward, including strengthening collaboration between hospitals, primary care centers, and community cadres, developing clear standard operating procedures (SOPs), ensuring accurate and complete patient data and providing training and leadership support for all professional groups.

*“We already collaborate with first-level facilities and cadres. If patients don’t come, cadres will visit them.”* (PM, F, 03)

*“As long as the SOPs are clear, this service can work well.”* (PD, F, 05)

Participants further emphasized that involving the community and families would strengthen patient adherence and continuity of TB care, both of which are considered essential for long‐term success.

### 3.4. Theme 4: Support for Telecare‐Based IPC

Participants noted the hospital’s robust infrastructure and commitment to piloting telecare services. The need for sustainable policies and active managerial support was identified as critical for the successful integration of these services into existing TB programs.

*“If you look at this telecare service, I think, in principle, we could do it here.”* (PD, F, 05)

The four themes and corresponding subthemes are summarized in Table 5 and illustrated in Figure 1, which depicts the relationships among opportunities, barriers, implementation strategies, and institutional support. Integration of quantitative and qualitative data indicated that healthcare workers generally recognized the value of IPC, although differences in perception and role clarity persisted across professions. Qualitative findings reinforced the quantitative results by highlighting organizational barriers and emphasizing the need for structured SOPs, clear task distribution, and digital readiness. Addressing gaps in professional collaboration and technological infrastructure is essential for the successful implementation of telecare for TB care in Indonesia.

## 4. Discussion

This study found that implementing a telecare‐based IPC model for TB management in an Indonesian hospital is promising but challenging. Healthcare workers acknowledged the importance of IPC, but their perceptions and professional roles differed significantly. The integration of telecare requires careful, coordinated preparation, especially regarding institutional policies, technological readiness, and human resources.

IPC is essential for holistic, patient‐centered TB care. In line with previous studies, this research found that doctors, nurses, and pharmacists have different views on collaboration, shaped by hierarchies, role ambiguity, and communication barriers. Doctors viewed coordination and goal‐setting more favorably, while nurses reported the greatest barriers, especially unclear responsibilities and limited recognition. These results reflect prior research showing that hierarchies and poor communication reduce IPC effectiveness and care quality [21–24].

Strengthening IPC requires shared goals, clear role delineation, and mutual respect among team members. Leadership within IPC teams should be dynamic—guided by expertise rather than hierarchy—to foster shared accountability and decision‐making. Developing structured communication pathways and continuous interprofessional education may enhance the understanding of roles and improve teamwork [25]. Effective IPC relies on positive perceptions, strong communication, and teamwork among healthcare professionals. Poor collaboration stems from miscommunication, differing education, lack of institutional support, and unclear role understanding [26–28]. Aligning perceptions through FGDs is essential to exploring telecare‐based IPC for TB patients. The FGD findings include the following.

Core competencies in implementing IPC practices include joint decision‐making, interprofessional values ​​and ethics, role clarification, communication, interprofessional conflict resolution, and reflection [29]. Each competency and related behavior aims to increase the expertise of all team members in creating and achieving goals, namely increasing compliance and therapeutic outcomes for TB patients. IPC will not work as it should if one of these four competencies does not work well [30].

IPC improves healthcare professionals’ knowledge and competence in managing drug‐resistant TB [31]. TB patients require long‐term support for prevention and treatment [32]. Multidisciplinary programs effectively address health issues holistically [30]. Health coaching motivates TB patients to adopt preventive behaviors, increasing awareness for patients and their families [33]. The second subtheme in the feasibility opportunity for telecare‐based IPC services is the similarity of perceptions regarding TB education among health workers (doctors, pharmacists, and nurses).

Telecare has the potential to enhance IPC by improving access, efficiency, and continuity of care for TB. The study highlighted opportunities such as prior collaboration, positive attitudes toward digital tools, and existing WhatsApp‐based systems. These findings align with global evidence that telecare, such as VDOT and mobile reminders, can improve adherence, lower costs, and broaden service reach in resource‐limited settings [34–36]. Hospital management demonstrated readiness to expand telecare, recognizing its potential for patient monitoring and counseling. Effective integration requires robust digital infrastructure, well‐defined protocols, and sustained institutional commitment. Telecare should complement, rather than replace, in‐person interactions, thereby supporting hybrid models of TB care.

Despite the positive outlook, several barriers were identified. These included task avoidance, unclear accountability, patient skepticism toward virtual communication, and the lack of SOPs. Similar challenges have been noted in other LMIC contexts. Limited digital literacy and inconsistent internet access impede telehealth adoption [37–39]. To address these barriers, organizations need clear leadership roles, training in interprofessional communication, and well‐developed SOPs. These actions will guide telecare implementation and ensure consistency across professional groups.

The TB treatment procedure consists of 2 stages, namely, intensive therapy for 2 months followed by a continuation phase of 4–7 months [40]. TB disease, which if in a good condition considers the disease has been cured. This can be an inhibiting factor in completing TB therapy [41]. Considering that patient compliance with treatment is one of the determining factors in the success of therapy, it is important to provide educational and counseling interventions to TB patients to prevent patient loss to follow‐up [42, 43].

Telecare enables remote TB treatment monitoring, reducing travel time, and offering flexibility. It is preferred to be accessible, affordable, and practical, particularly in underserved areas, by minimizing side effects, improper medication use, and costs [17, 27, 35–37, 39].

A prior study at this hospital revealed that most patients were open to the concept of telecare services. Optimism and innovativeness were identified as key factors affecting patients’ readiness to engage with telecare. TB patients also expressed positive views regarding the system’s ease of use and its perceived advantages [18].

Building on these findings, the current study aimed to explore the opportunities, challenges, and supporting elements influencing the implementation of a telecare‐based IPC model for TB patients. Results from the FGDs showed that the successful application of telecare‐based IPC depends on clearly structured service pathways and thorough preparation at three levels: patients, hospitals, and healthcare teams.

From the patient perspective, readiness involves outpatient TB patients who are willing to participate in telecare‐based IPC, have access to supporting tools such as smartphones, and are at risk of nonadherence, particularly those with comorbidities. From the hospital’s perspective, preparation includes ensuring the availability of telecare infrastructure such as WhatsApp Business and broadcast systems, along with budget allocation for the program’s execution. From the healthcare team’s standpoint, readiness requires commitment and competence among pulmonologists, pharmacists, and nurses capable of effective interprofessional communication and collaboration.

Both previous studies and FGDs indicate that telecare‐based IPC is achievable when supported by appropriate strategies. Digital health tools, such as VOT, have been shown to improve TB treatment adherence and outcomes [34, 44]. Collaboration among doctors, nurses, pharmacists, and social workers provides comprehensive support that enhances adherence and improves quality of life [45].

The findings indicate that the success of telecare‐based IPC depends on the readiness of patients, hospitals, and healthcare teams. Patients require access to digital tools and a willingness to engage in telecare. Hospitals must invest in infrastructure and offer policy support. Healthcare teams need to collaborate effectively, communicate clearly, and uphold ethical standards. These insights inform the development of scalable, patient‐centered telecare models that align with the WHO End TB Strategy.

### 4.1. Strength and Limitations

The strengths of this study include its mixed‐methods design, involvement of multidisciplinary participants, and validation through triangulation and member checking. However, the findings are limited to healthcare providers at a single institution, which may restrict generalizability. Future research should incorporate patient and community perspectives, evaluate telecare‐based IPC in diverse settings, and assess its effects on treatment adherence and clinical outcomes.

### 4.2. Study Implications

TB is still an unresolved problem, especially in Indonesia. The ongoing program still shows a gap between targets and achievements. Service innovation is needed to accelerate the elimination of TB in line with the end TB target of 2035. The development of telecare‐based IPC services can address the complexity of TB treatment, particularly in diverse population characteristics, high‐risk patients, communities with a high prevalence of TB, and areas with good access to health services, such as Surabaya City.

## 5. Conclusion

This study demonstrates that telecare‐based IPC is both feasible and effective in supporting TB management by facilitating coordinated, efficient, and patient‐centered care. The primary finding is the model’s potential to strengthen TB control efforts significantly. Critical success factors include institutional readiness, strong infrastructure, and collective professional commitment. Although the model is adaptable, local customization is necessary to optimize its impact on Indonesia’s TB elimination initiatives and advance the global End TB Strategy.

## Funding

This study received funding from the Universitas Airlangga—Management and Science University Research Collaboration Grant, 684/UN3.15/PT/2023.

## Conflicts of Interest

The authors declare no conflicts of interest.

## Data Availability

The data that support the findings of this study are available on request from the corresponding authors. The data are not publicly available due to privacy or ethical restrictions.
